# Supplementary material for: Direct control of lysosomal catabolic activity by mTORC1 through regulation of V-ATPase assembly
Source: Nat Commun. 2022 Aug 17;13:4848. doi: 10.1038/s41467-022-32515-6 (PMC9385660; doi:10.1038/s41467-022-32515-6)
Supplement: Supplementary file 1 — Supplementary Information [file 41467_2022_32515_MOESM1_ESM.pdf]

Supplementary Information:

Direct control of lysosomal catabolic activity by mTORC1 through regulation of V-ATPase assembly

Edoardo Ratto[1,2], S. Roy Chowdhury[1], Nora S. Siefert[1], Martin Schneider[3], Marten Wittmann[1], Dominic Helm[3], Wilhelm Palm[1*]

[1]Cell Signaling and Metabolism, German Cancer Research Center (DKFZ), Heidelberg, Germany

[2]Faculty of Biosciences, University of Heidelberg, Heidelberg, Germany

[3]MS-based Protein Analysis Unit, Genomics and Proteomics Core Facility, German Cancer Research Center (DKFZ), Heidelberg, Germany

[*] To whom correspondence should be addressed: w.palm@dkfz-heidelberg.de

Supplementary figures

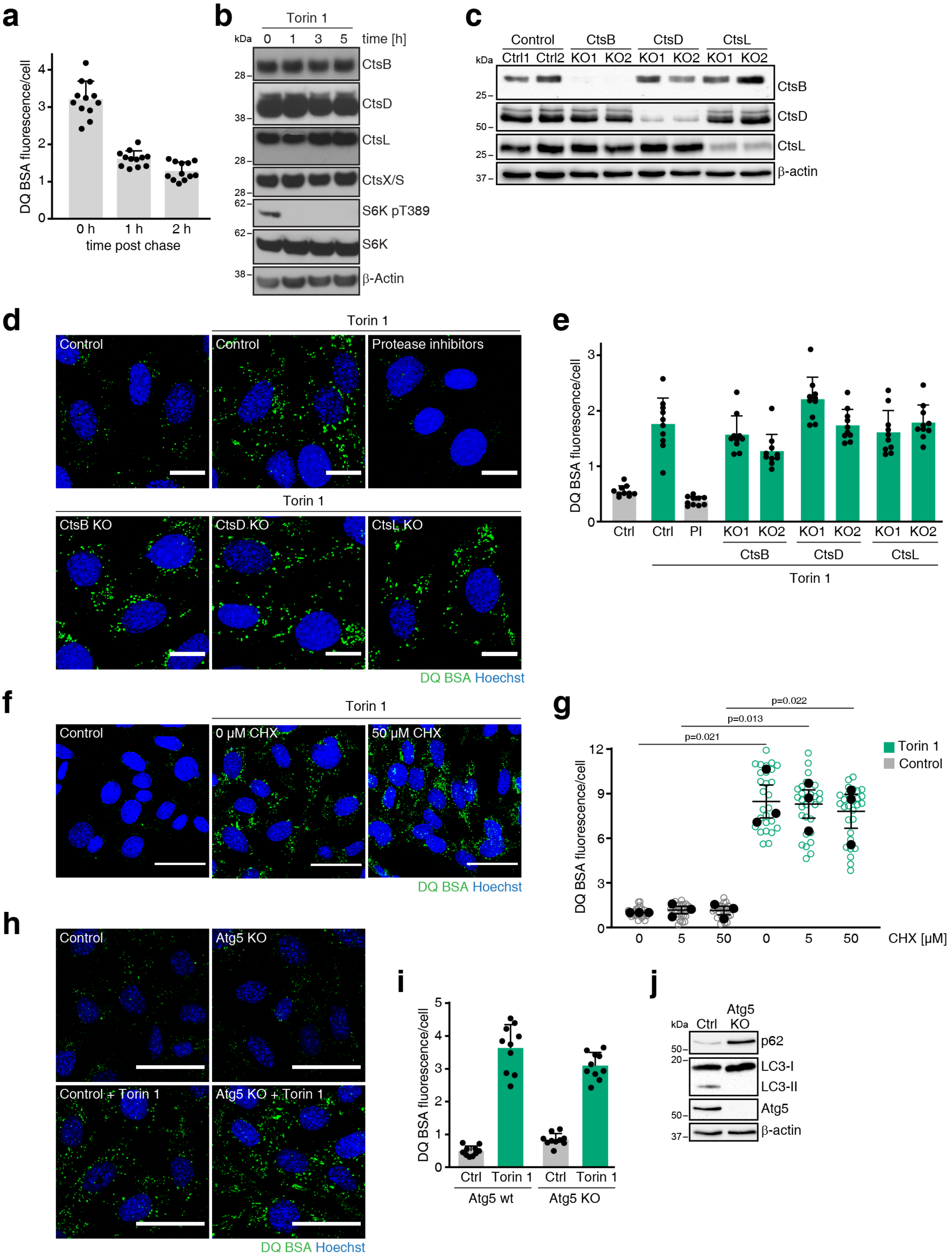

Supplementary Figure 1 mTORC1 does not block lysosomal catabolism of extracellular proteins through changes in cathepsin levels, protein synthesis or autophagy

a) Quantification of DQ BSA degradation in MEFs after 4 h pre-loading followed by chase for indicated periods of time. Data are mean ± SD (10 fields of view). **b)** Mature cathepsin levels in MEFs + torin 1 [250 nM] for indicated periods of time, analysed by western blot. **c)** CRISPR/Cas9-mediated deletion of cathepsin B, cathepsin D or cathepsin L, analysed by western blot. **d)** DQ BSA degradation in MEFs deficient for cathepsin B, cathepsin D or cathepsin L, or treated with protease inhibitors (PI; 20 µM pepstatin, leupeptine, E64, AEBSF) after 5 h DQ BSA uptake + torin 1 [400 nM]. Scale bars = 20 µm. **e)** Quantification of DQ BSA fluorescence of cells shown in d). Data are mean ± SD (10 fields of view). **f)** DQ BSA degradation in MEFs after 5 h DQ BSA uptake ± torin 1 [250 nM] and cycloheximide (CHX) at indicated concentrations. Scale bars = 50 µm. **g)** Quantification of DQ BSA fluorescence of cells treated as in f). Data are normalized replicate mean ± SEM (closed circles) and fields of view (open circles; 6-12 per replicate). **h)** DQ BSA degradation in Atg5-deficient MEFs after 5 h DQ BSA uptake ± torin 1 [400 nM]. Scale bars = 20 µm. **i)** Quantification of DQ BSA fluorescence of cells shown in h). Data are mean ± SD (10 fields of view). **j)** CRISPR/Cas9-mediated deletion of Atg5, analysed by western blot. a), e), i) One representative of n=3 biologically independent experiments. b), c), j) One representative of n=2 biologically independent experiments. g) n=3 biologically independent experiments. p-values were calculated using a two-tailed unpaired t-test with Welch correction. Source data are provided as a Source Data file.

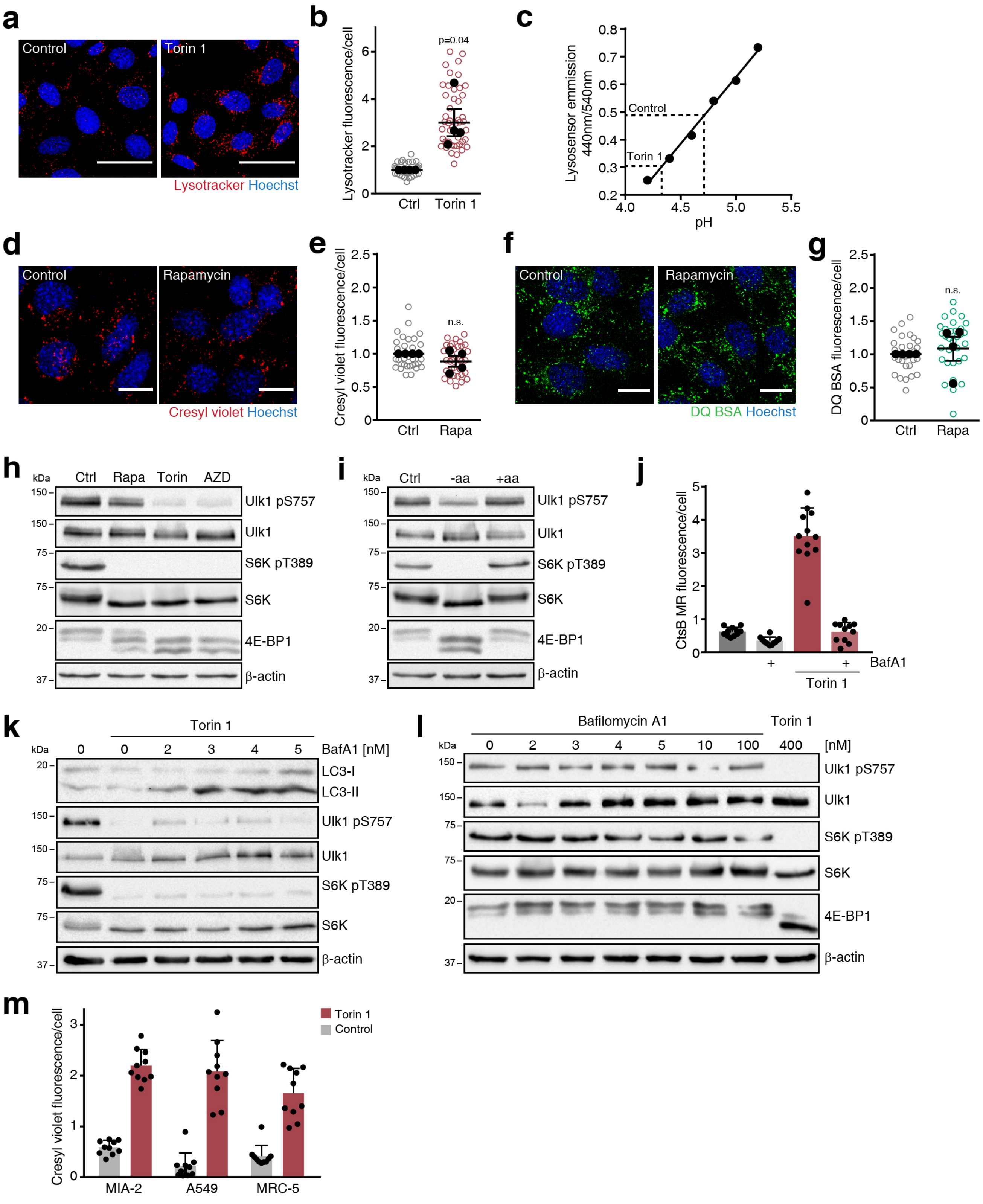

a
Control
Torin 1
Lysotracker Hoechst
b
Lysotracker fluorescence/cell
p=0.04
Ctrl
Torin 1
c
Lysosensor emmission 440nm/540nm
Control
Torin 1
pH
d
Control
Rapamycin
Cresyl violet Hoechst
e
Cresyl violet fluorescence/cell
n.s.
Ctrl
Rapa
f
Control
Rapamycin
DQ BSA Hoechst
g
DQ BSA fluorescence/cell
n.s.
Ctrl
Rapa
h
Ctrl Rapa Torin AZD
Ulk1 pS757
Ulk1
S6K pT389
S6K
4E-BP1
β-actin
i
Ctrl -aa +aa
Ulk1 pS757
Ulk1
S6K pT389
S6K
4E-BP1
β-actin
j
CtsB MR fluorescence/cell
BafA1
Torin 1
k
Torin 1
BafA1 [nM]
LC3-I
LC3-II
Ulk1 pS757
Ulk1
S6K pT389
S6K
β-actin
l
Bafilomycin A1
Torin 1
[nM]
Ulk1 pS757
Ulk1
S6K pT389
S6K
4E-BP1
β-actin
m
Cresyl violet fluorescence/cell
Torin 1
Control
MIA-2
A549
MRC-5

Supplementary Figure 2 mTORC1 regulates lysosomal catabolism

a) Lysotracker accumulation in MEFs after 1 h ± torin 1 [400 nM]. Scale bars = 50 µm. **b)** Quantification of lysotracker fluorescence in cells treated as in a). Data are normalized replicate mean ± SEM (closed circles) and fields of view (open circles; 10-15 per replicate). **c)** Exemplary calibration curve for lysosensor-based pH measurements. Dashed lines indicate dual-emission ratiometric measurements and corresponding pH values in MEFs after 1 h ± torin 1 [400 nM] in the same experiment. **d)** Cresyl violet accumulation in MEFs after 1 h ± rapamycin [200 nM]. Scale bars = 20 µm. **e)** Quantification of cresyl violet fluorescence in cells treated as in d). Data are normalized replicate mean ± SEM (closed circles) and fields of view (open circles; 8-10 per replicate). **f)** DQ-BSA degradation in MEFs after 5 h DQ BSA uptake ± rapamycin [200 nM]. Scale bars = 20 µm. **g)** Quantification of DQ BSA degradation in cells treated as in f). Data are normalized replicate mean ± SEM (closed circles) and fields of view (open circles; 6-10 per replicate). **h)** mTORC1 signalling activity in MEFs after 1 h rapamycin [100 nM], torin 1 [250 nM], or AZD8055 [250 nM] as in Fig. 2c), analysed by western blot. **i)** mTORC1 signalling activity in MEFs after 1 h amino acid starvation (-aa), or 1 h aa starvation + 30 min aa restimulation (+aa) as in Fig. 2d), analysed by western blot. **j)** Quantification of cathepsin B magic red substrate degradation in MEFs pre-treated ± 1 h torin 1 [250 nM], bafilomycin A1 [2.5 nM]. Data are mean ± SD (10 fields of view). **k)** LC3-II levels and mTORC1 signalling activity in MEFs after 2 h + torin 1 [400 nM] and bafilomycin A1 at indicated concentrations, analysed by western blot. **l)** mTORC1 signalling activity in MEFs after 2 h + bafilomycin A1 at indicated concentrations or torin 1 [400 nM], analysed by western blot. **m)** Quantification of cresyl violet accumulation after 3 h ± torin 1 [400 nM] in cell lines shown in Fig. 2h). Data are mean ± SD (10 fields of view). b), e), g) n=4 biologically independent experiments. h) - m) One representative of n=3 biologically independent experiments. p-values were calculate using a two-tailed unpaired t-test with Welch correction. n.s. not significant. Source data are provided as a Source Data file.

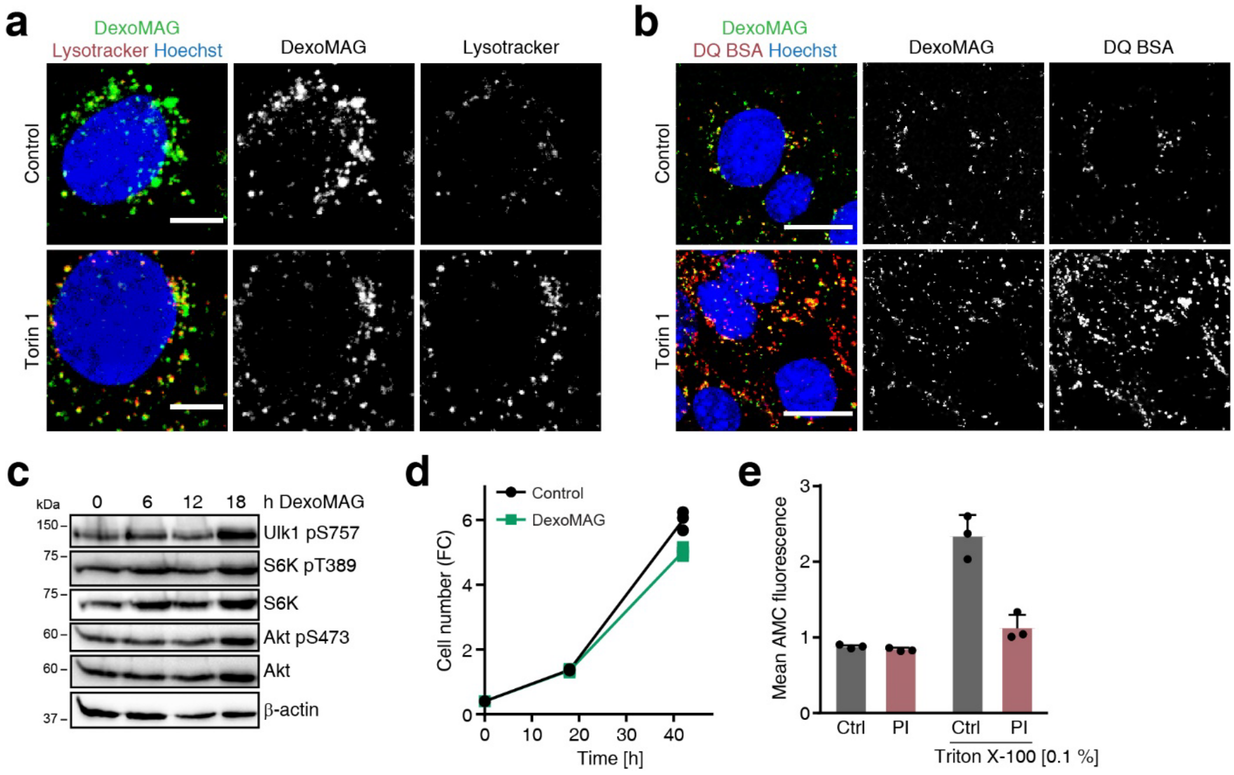

Supplementary Figure 3 Quality controls for magnetic enrichment of lysosomes

a) Co-localization of fluorescent DexoMAG and lysotracker in MEFs ± torin 1 [250 nM]. Scale bars = 10 µm. **b)** Co-localization of fluorescent DexoMAG and DQ BSA in MEFs after 5 h ± torin 1 [250 nM]. Scale bars = 20 µm. **c)** mTORC1 signalling activity in MEFs after incubation with DexoMAG for indicated periods of time, analysed by western blot. **d)** Proliferation of MEFs ± DexoMAG. Data are mean ± SD (3 technical replicates). **e)** Integrity of magnetically enriched lysosomes, as assessed by fluorescence dequenching of the cathepsin B substrate Z-Arg-Arg-AMC. Note that cathepsin activity increases substantially upon permeabilization of lysosomes with 0.1 % Triton X-100. PI: protease inhibitor (10 µM leupeptin). Data are mean ± SD (3 technical replicates). a) - e) One representative of n=2 biologically independent experiments. Source data are provided as a Source Data file.

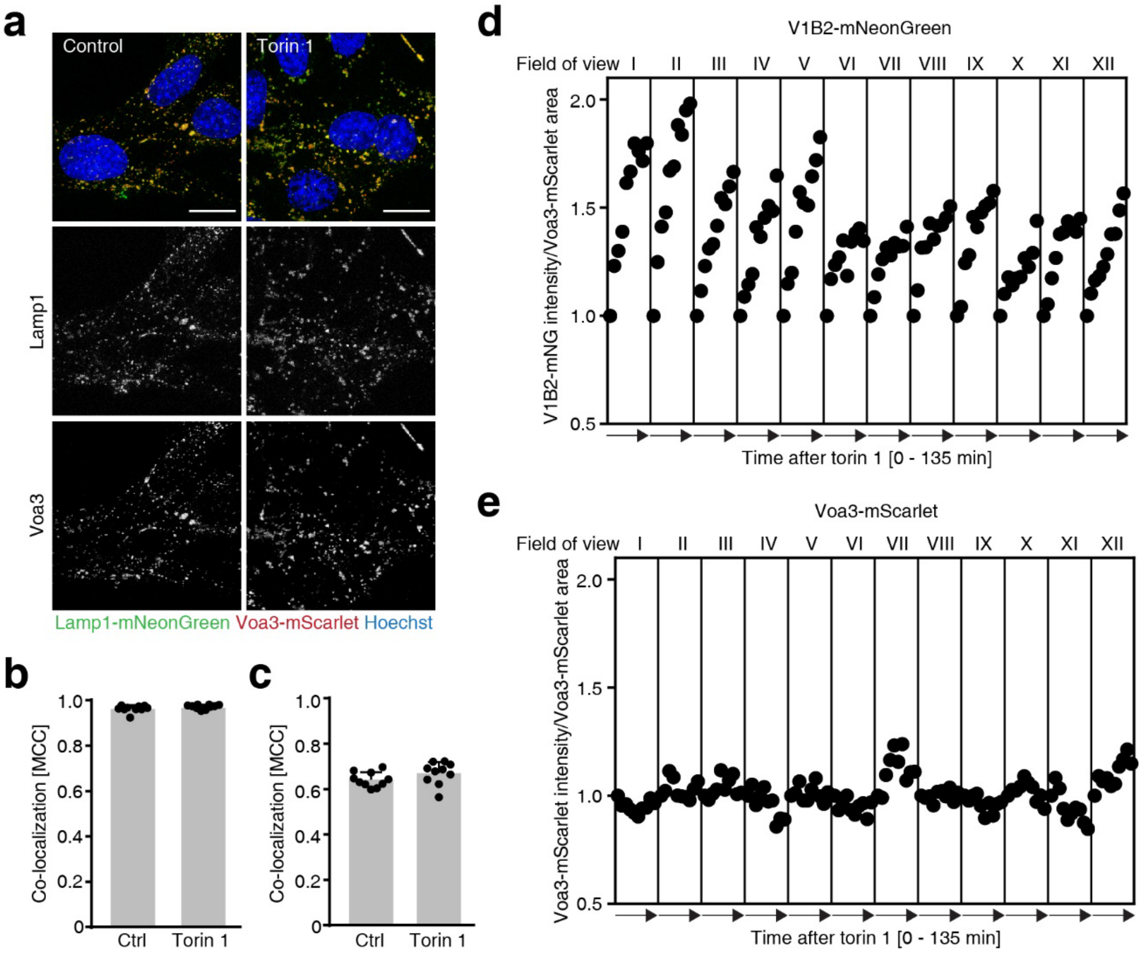

Supplementary Figure 4 Live cell imaging reveals regulation of V-ATPase assembly at lysosomes by mTORC1

a) Co-localization of Voa3-mScarlet with Lamp1-mNeonGreen in MEFs after 1 h ± torin 1 [400 nM]. Scale bars = 20 µm. **b)**, **c)** Manders correlation coefficient (MCC) for co-localization of b) Voa3-mScarlet with Lamp1-mNeonGreen, c) Lamp1-mNeonGreen with Voa3-mScarlet of cells shown in a). Data are mean ± SD (10 fields of view). **d)**, **e)** Quantification of d) V1B2-mNeonGreen levels in Voa3-mScarlet-containing organelles, e) organellar Voa-3-mScarlet levels after torin 1 treatment [400 nM] over time. Data are individual fields of view in 15 min intervals. See also Fig. 4c). d), e) One representative of n=3 biologically independent experiments. Source data are provided as a Source Data file.

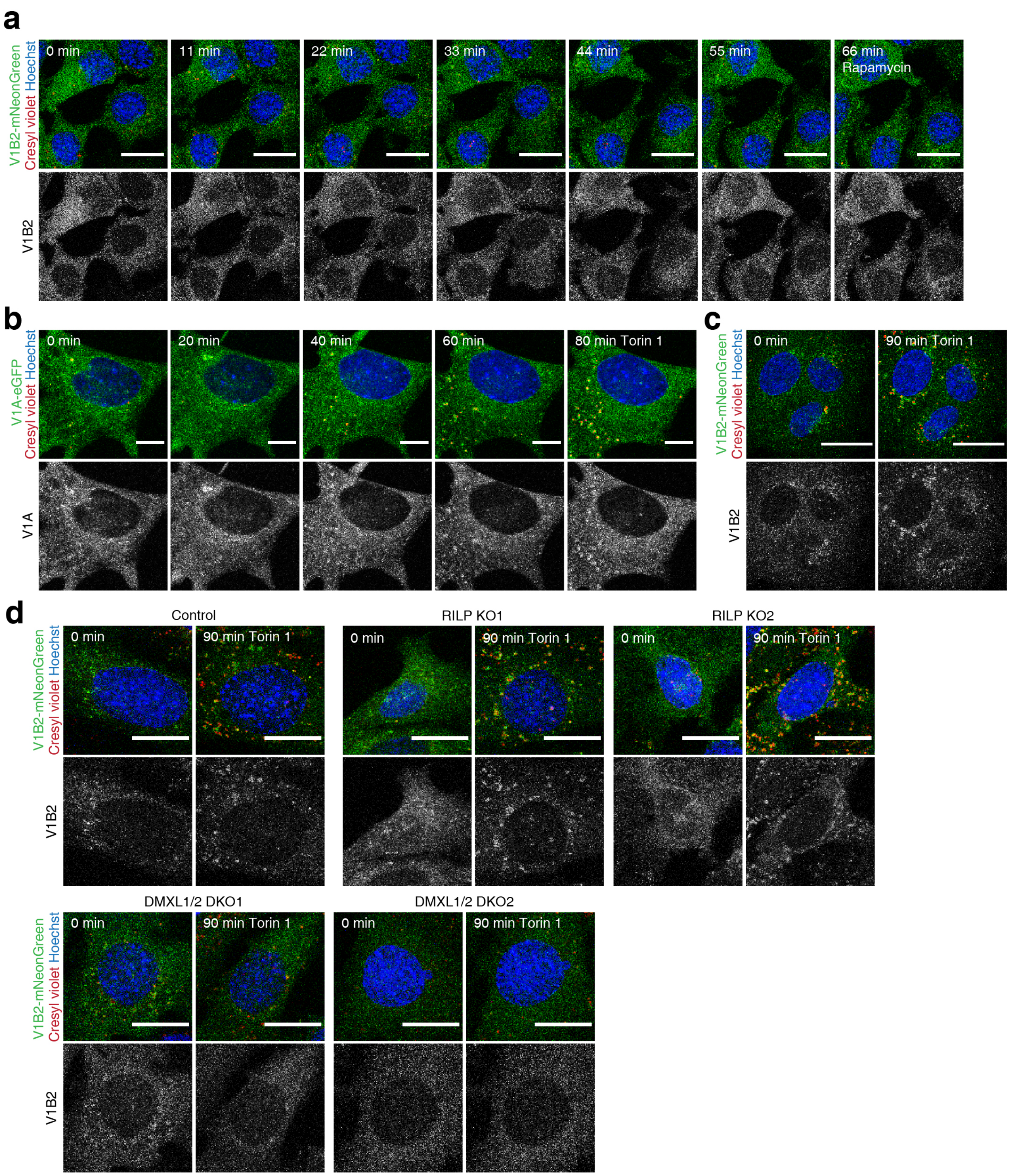

a
V1B2-mNeonGreen Cresyl violet Hoechst
0 min
11 min
22 min
33 min
44 min
55 min
66 min Rapamycin
V1B2
b
V1A-eGFP Cresyl violet Hoechst
0 min
20 min
40 min
60 min
80 min Torin 1
V1A
c
V1B2-mNeonGreen Cresyl violet Hoechst
0 min
90 min Torin 1
V1B2
d
Control
RILP KO1
RILP KO2
V1B2-mNeonGreen Cresyl violet Hoechst
0 min
90 min Torin 1
V1B2
DMXL1/2 DKO1
DMXL1/2 DKO2

Supplementary Figure 5 Lysosomal movement of V-ATPase V_1 domains and lysosomal acidification in response to mTORC1 inactivation

a) No lysosomal accumulation of V1B2-mNeonGreen and cresyl violet in MEFs over time upon treatment with rapamycin [200 nM]. Scale bars = 20 µm. **b)** Lysosomal accumulation of V1A-eGFP and cresyl violet in MEFs over time upon treatment with torin 1 [400 nM]. Scale bars = 10 µm. **c)** Lysosomal accumulation of V1B2-mNeonGreen and cresyl violet in A549 cells before and after 90 min + torin 1 [400 nM]. Scale bars = 20 µm. **d)** Changes in lysosomal accumulation of V1B2-mNeonGreen and cresyl violet in MEFs deficient for RILP or DMXL1/2 before and after 90 min + torin 1 [400 nM]. Scale bars = 20 µm. a) - d) One representative of n=3 biologically independent experiments.

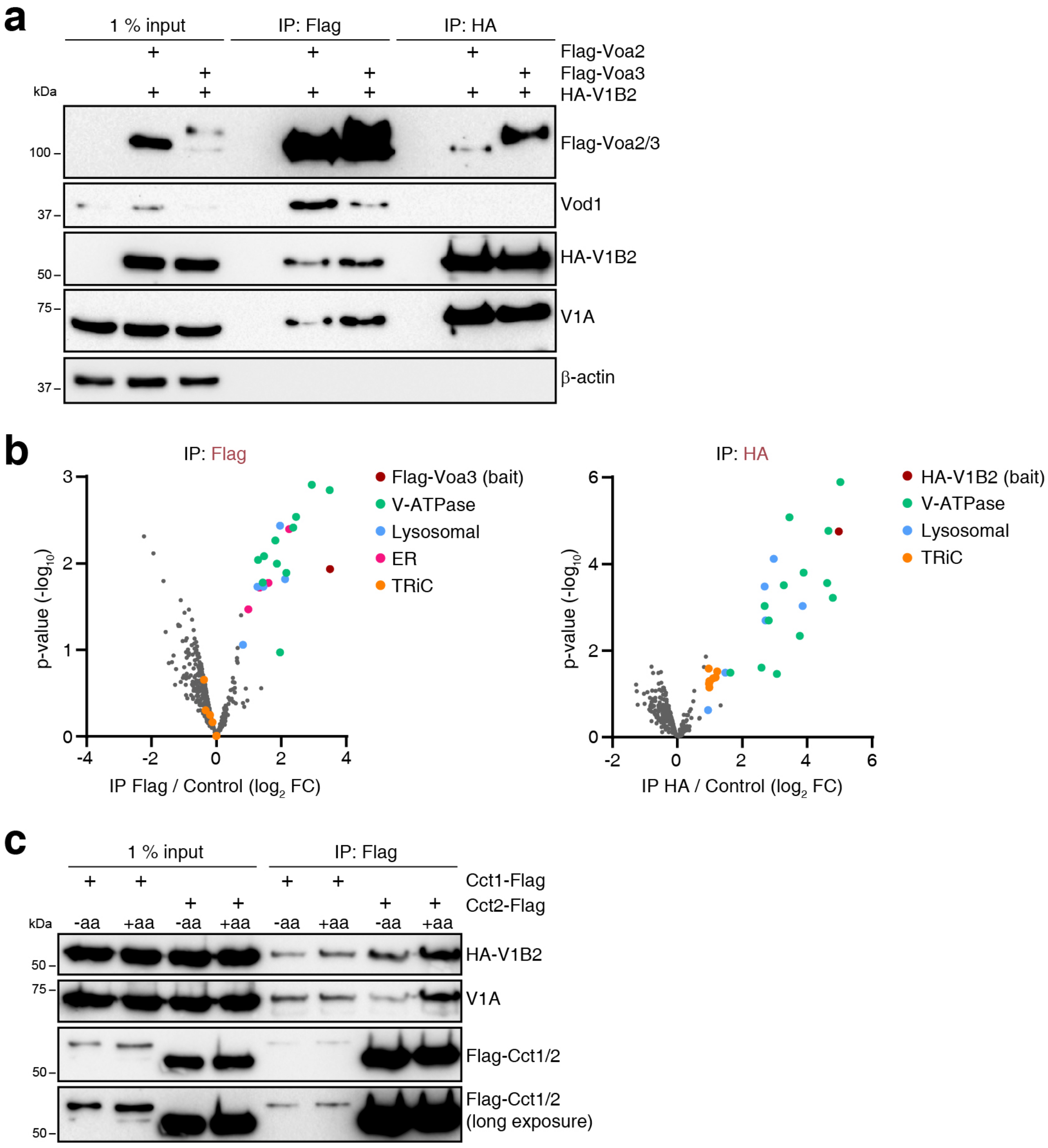

Supplementary Figure 6 The chaperonin TRiC reversibly associates with the V-ATPase V_1 domain

a) Co-IP of V-ATPase subunits in MEFs by Flag-Voa3, Flag-Voa2 or HA-V1B2, analysed by western blot. **b)** Co-IP – SILAC proteomics of V-ATPase subcomplex-interacting proteins. Shown is the $\log_2$ fold change of proteins from cells expressing the respective bait (Flag-Voa3 or HA-V1B2) over vector control cells. **c)** Increased Co-IP of V-ATPase V_1 domain subunits in MEFs with TRiC subunits Cct1 or Cct2 after 1 h amino acid starvation + 30 min amino acid restimulation (+aa) and after 1 h amino acid starvation (-aa), analysed by western blot. a) One representative of n=3 biologically independent experiments. b) n=4 biologically independent experiments. p-values were calculated using limma moderated t-statistic. c) One representative of n=2 biologically independent experiments. Source data are provided as a Source Data file.

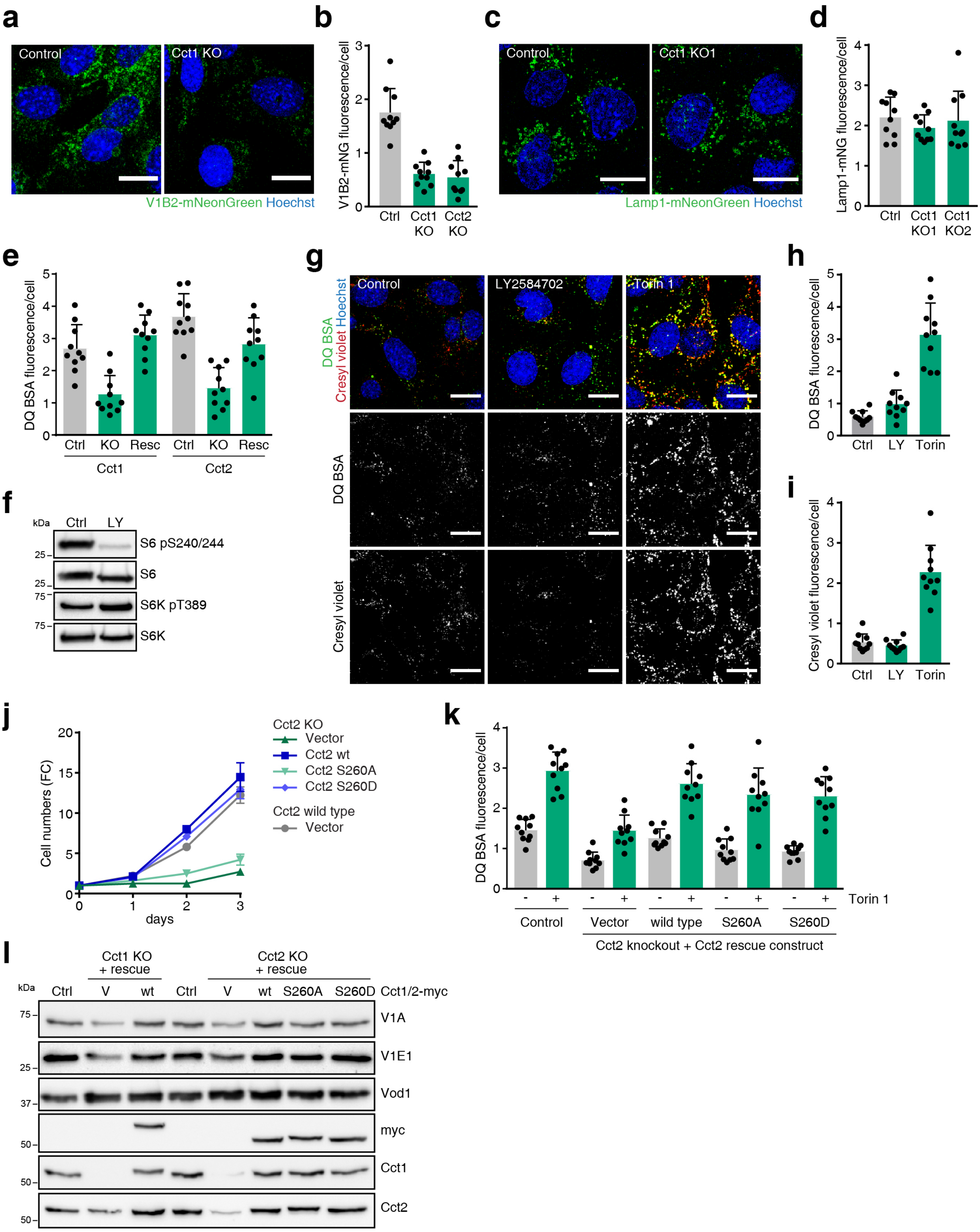

a
Control
Cct1 KO
V1B2-mNeonGreen Hoechst
b
V1B2-mNG fluorescence/cell
Ctrl
Cct1 KO
Cct2 KO
c
Control
Cct1 KO1
Lamp1-mNeonGreen Hoechst
d
Lamp1-mNG fluorescence/cell
Ctrl
Cct1 KO1
Cct1 KO2
e
DQ BSA fluorescence/cell
Ctrl
KO
Resc
Cct1
Cct2
f
kDa
Ctrl
LY
S6 pS240/244
S6
S6K pT389
S6K
g
DQ BSA Cresyl violet Hoechst
Control
LY2584702
Torin 1
DQ BSA
Cresyl violet
h
DQ BSA fluorescence/cell
Ctrl
LY
Torin
i
Cresyl violet fluorescence/cell
Ctrl
LY
Torin
j
Cell numbers (FC)
days
Cct2 KO
Vector
Cct2 wt
Cct2 S260A
Cct2 S260D
Cct2 wild type
Vector
k
DQ BSA fluorescence/cell
Torin 1
Control
Vector
wild type
S260A
S260D
Cct2 knockout + Cct2 rescue construct
l
Cct1 KO + rescue
Cct2 KO + rescue
kDa
Ctrl
V
wt
S260A
S260D
Cct1/2-myc
V1A
V1E1
Vod1
myc
Cct1
Cct2

Supplementary Figure 7 Stabilization of the V-ATPase V_1 domain by TRiC is not regulated by S6K-mediated phosphorylation of Cct2

a) V1B2-mNeonGreen levels in Cct1-deficient MEFs. Scale bars = 20 µm. **b)** Quantification of V1B2-mNeonGreen levels in Cct1 and Cct2-deficient MEFs. **c)** Lamp1-mNeonGreen levels in Cct1-deficient MEFs. Scale bars = 20 µm. **d)** Quantification of Lamp1-mNeonGreen levels in Cct1-deficient MEFs. **e)** Quantification of DQ BSA degradation in Cct1 and Cct2-deficient MEFs ectopically expressing Cct1 and Cct2 rescue construct, respectively, after 5 h DQ BSA uptake + torin 1 [400 nM]. **f)** Inhibition of S6K activity after 6 h LY2584702 [5 µM], analysed by western blot. **g)** DQ BSA and cresyl violet in MEFs after 5 h + LY2584702 [5 µM] or torin 1 [400 nM]. Scale bars = 20 µm. **h)**, **i)** Quantification of h) DQ BSA, i) cresyl violet in MEFs after 5 h DQ BSA uptake + LY2584702 [5 µM] or torin 1 [400 nM] as shown in g). **j)** Proliferation of Cct2-deficient MEFs ectopically expressing Cct2 wild type, S260A or S260D. **k)** Quantification of DQ BSA degradation in Cct2-deficient MEFs ectopically expressing Cct2 wild type, S260A or S260D after 5 h DQ BSA uptake ± torin 1 [400 nM]. **l)** Changes in V-ATPase subunit abundance in Cct1 and Cct2-deficient MEFs ectopically expressing Cct1 and Cct2 rescue constructs, respectively, analysed by western blot. b), d), e), h), i), k) Data are mean ± SD (10 fields of view). j) Data are mean ± SD (3 technical replicates). a) - i) one representative of n=3 biologically independent experiments. k), l) one representative of n=5 biologically independent experiments. Source data are provided as a Source Data file.

Supplementary Tables

Supplementary Table 1 sgRNA sequences

Gene	Species	Name	sgRNA sequence 1	sgRNA sequence 2
**Chr1.1**	mouse	Control_sgRNA1	GACAATGAACATAAGCACAT	
**Rosa1**	mouse	Control_sgRNA2	GAAGATGGGCGGGAGTCTTC	
**Cct1**	mouse	CCT1_sgRNA1	GGTGGCACCATCGTTAGTAA	
**Cct2**	mouse	CCT2_sgRNA1	GGTTGGAGAGAAGCCACAA	
**Atg5**	mouse	ATG5_dsgRNA	ATCAAATAGTAAACCAAT	GAACATCACAGTACATTTCA
**Rilp1**	mouse	RILP1_dsgRNA1	TCTTGGAAAAGGCCGCCGTG	GCTCGTGTACCATCTAGCGG
**Rilp1**	mouse	RILP1_dsgRNA2	GGAGGTGACAGACAGACAGC	TCTTGGAAAAGGCCGCCGTG
**Dmxl1**	mouse	DMXL1_dsgRNA1	AGTTGGGGAAAGTACATGAG	TGATGGTGAGAGATCTAAGG
**Dmxl1**	mouse	DMXL1_dsgRNA2	AGTAGGGAGCCATGCCACAT	GCATTAAGGACACCAAATGT
**Dmxl2**	mouse	DMXL2_dsgRNA1	GTGGGACACAGTCAGAACAG	TATATGACAAAGGTCCAATG
**Dmxl2**	mouse	DMXL2_dsgRNA2	GGTAAGAGATGGAATATTGG	TTGAATGCGAATCTACAGGA
**CtsB**	mouse	CTSB_dsgRNA1	GGAGTCTACAATTCTCATGT	CTGGAGAAGGAGATACTCCC
**CtsB**	mouse	CTSB_dsgRNA2	TCGGCCATTGGTGTGAATGC	ATTGGACAGATTAGAGACCA
**CtsD**	mouse	CTSD_dsgRNA1	GTATCTTGCAATGAATGGAG	CCTGAGCCAGGACACTGTAT
**CtsD**	mouse	CTSD_dsgRNA2	GGGGCAGTGCCTCTTATCCA	CACTCGAAAGGCCTACTGGC
**CtsL**	mouse	CTSL_dsgRNA1	GCAAGAGAAAGCCCTCATGA	GGACAGATGTTCCTTAAGAC
**CtsL**	mouse	CTSL_dsgRNA2	AAGAGTGGAGGAGAGCGATA	CCAAGCAGAGGACAGCCAAA
**Cct1**	mouse	CCT1_dsgRNA1	AGTTGGCTTGGATAAAATGT	GTTCGGGGACCGCAGCACTG
**Cct1**	mouse	CCT1_dsgRNA2	GTTCGGGGACCGCAGCACTG	CAGATCATCAATCCGAAGGA
**Cct2**	mouse	CCT2_dsgRNA1	AGTGGCACCACGAAGCACAA	GATCCTAGAACAGTTTACGG

Supplementary Table 2 PCR primers for endogenous tagging of Lamp1

Primer ID	Sequence (5' to 3')
**M1_mLAMP1**	G*T*G*G*G*CGGTGCCCTGGCAGGGCTGGTCCTCATCGTCCTCATTGCCTACCTCATTGGC AGGAAGAGGAGTCACGCCGGCTATCAGACCATCTCAGGTGGAGGAGGTAGTG
**M2_mLAMP1**	G*C*T*T*G*GGGATGTGAGAACAGGCCCCTGTGCATCTCTGGTGCACCTGCCCACCAGGAA AAAACCCACCAGGCTAGATGGTCTATCTACAAGAGTAGAAATTAGCTAGCTGCATCGGTA CC
