# Supplementary material for: Direct control of lysosomal catabolic activity by mTORC1 through regulation of V-ATPase assembly
Source: Nat Commun. 2022 Aug 17;13:4848. doi: 10.1038/s41467-022-32515-6 (PMC9385660; doi:10.1038/s41467-022-32515-6)
Supplement: Supplementary file 3 — Description of Additional Supplementary Files [file 41467_2022_32515_MOESM3_ESM.pdf]

Description of Additional Supplementary Files

Supplementary Data 1 Proteomics datasets for enriched lysosomal fractions

Table 1: Quantitative proteomics of post-nuclear supernatants (PNS) and magnetically enriched lysosomes (eluates) from MEFs. Log2 fold-changes, p-values and adjusted p-values of imputed values for lysosomal fractions compared to post-nuclear supernatants (PNS) are provided. Table 2: Quantitative proteomics of enriched lysosomes (eluates) from MEFs after 1 h amino acid starvation + 30 min amino acid restimulation compared to 1 h amino acid starvation. Log2 fold-changes, p-values and adjusted p-values of imputed values are provided. For each of the 5 replicates, the number of unique peptides is listed.

Supplementary Data 2 Proteomics datasets for SILAC-Co-IPs

Table 1: Quantitative proteomics of Flag Co-IPs and HA Co-IPs from MEFs expressing Flag-Voa3 and HA-V1B2, respectively, compared to vector control MEFs. Log2 fold-changes, p-values and adjusted p-values of imputed values are provided. Table 2: Quantitative proteomics of Co-IPs from MEFs expressing Flag-Voa3 or HA-V1B2 after 1 h amino acid starvation compared to full medium (-aa), or after 1 h amino acid starvation + 30 min amino acid restimulation compared to 1 h amino acid starvation (+aa). Log2 fold-changes, p-values and adjusted p-values of imputed values are provided. For each of the 4 replicates, the number of unique peptides is listed.

Supplementary Movie 1 Live cell imaging of V1B2-mNeonGreen upon torin 1 treatment

MEFs expressing V1B2-mNeonGreen were treated with torin 1 [400 nM] at the onset of the movie.

Supplementary Movie 2 Live cell imaging of V1B2-mNeonGreen after amino acid restimulation

MEFs expressing V1B2-mNeonGreen were subjected to 1 h amino acid starvation, followed by amino acid restimulation at the onset of the movie.
