# Supplementary material for: Direct control of lysosomal catabolic activity by mTORC1 through regulation of V-ATPase assembly
Source: Nat Commun. 2022 Aug 17;13:4848. doi: 10.1038/s41467-022-32515-6 (PMC9385660; doi:10.1038/s41467-022-32515-6)
Supplement: Supplementary file 8 — Reporting Summary [file 41467_2022_32515_MOESM8_ESM.pdf]

nature portfolio

Corresponding author(s): Wilhelm Palm

Last updated by author(s): Jul 13, 2022

Reporting Summary

Nature Portfolio wishes to improve the reproducibility of the work that we publish. This form provides structure for consistency and transparency in reporting. For further information on Nature Portfolio policies, see our Editorial Policies and the Editorial Policy Checklist.

Statistics

For all statistical analyses, confirm that the following items are present in the figure legend, table legend, main text, or Methods section.

n/a	Confirmed	
☐	☒	The exact sample size (*n*) for each experimental group/condition, given as a discrete number and unit of measurement
☐	☒	A statement on whether measurements were taken from distinct samples or whether the same sample was measured repeatedly
☐	☒	The statistical test(s) used AND whether they are one- or two-sided<br>*Only common tests should be described solely by name; describe more complex techniques in the Methods section.*
☒	☐	A description of all covariates tested
☐	☒	A description of any assumptions or corrections, such as tests of normality and adjustment for multiple comparisons
☐	☒	A full description of the statistical parameters including central tendency (e.g. means) or other basic estimates (e.g. regression coefficient) AND variation (e.g. standard deviation) or associated estimates of uncertainty (e.g. confidence intervals)
☐	☒	For null hypothesis testing, the test statistic (e.g. *F*, *t*, *r*) with confidence intervals, effect sizes, degrees of freedom and *P* value noted<br>*Give P values as exact values whenever suitable.*
☒	☐	For Bayesian analysis, information on the choice of priors and Markov chain Monte Carlo settings
☒	☐	For hierarchical and complex designs, identification of the appropriate level for tests and full reporting of outcomes
☒	☐	Estimates of effect sizes (e.g. Cohen's *d*, Pearson's *r*), indicating how they were calculated

Our web collection on statistics for biologists contains articles on many of the points above.

Software and code

Policy information about availability of computer code

Data collection	FACS: BD FACSDiva software (v8.0, FACSAria I).<br>Microscopy: Leica LAS AF software (v2.6.3.8173)<br>Western blotting: ChemiDoc Touch imaging system (BioRad v6.0.0)<br>Plate Reader: Multi-Mode Plate Reader Synergy H1 (BioTek).
Data analysis	Statistical analyses were performed with GraphPad Prism version 9.0.0 (471).<br>Wester Blot quantification: Image Lab software (v3.0.1.14).<br>Image analysis: ImageJ / FIJI (v1.52n).<br>Data compiling, processing and statistical analyses: GraphPad Prism 9 (v9.0.0)<br>Proteomics data analysis was performed with MaxQuant (v1.6.14.0) and the R-package limma (v3.44.3).

For manuscripts utilizing custom algorithms or software that are central to the research but not yet described in published literature, software must be made available to editors and reviewers. We strongly encourage code deposition in a community repository (e.g. GitHub). See the Nature Portfolio guidelines for submitting code & software for further information.

March 2021

Data

Policy information about availability of data

All manuscripts must include a data availability statement. This statement should provide the following information, where applicable:

- Accession codes, unique identifiers, or web links for publicly available datasets
- A description of any restrictions on data availability
- For clinical datasets or third party data, please ensure that the statement adheres to our policy

The proteomics data generated in this study have been deposited at ProteomeXchange: PXD030172 [https://www.ebi.ac.uk/pride/archive/projects/PXD030172], PXD030174 [https://www.ebi.ac.uk/pride/archive/projects/PXD030174]. Other data generated in this study are provided in Supplementary Data files 1 and 2, and in the Supplementary Information and Source Data files.

Field-specific reporting

Please select the one below that is the best fit for your research. If you are not sure, read the appropriate sections before making your selection.

☒ Life sciences ☐ Behavioural & social sciences ☐ Ecological, evolutionary & environmental sciences

For a reference copy of the document with all sections, see nature.com/documents/nr-reporting-summary-flat.pdf

Life sciences study design

All studies must disclose on these points even when the disclosure is negative.

Sample size	For cell culture-based experiments, sample sizes were determined based on pilot experiments and previous experience with the models and the methods used in this study: DOI: 10.1016/j.cell.2015.06.017
Data exclusions	In few instances, western blots were removed due to blotting artifacts and fields of views due microscope stage mechanical problems.
Replication	The number of replicates in each experiment is detailed in the figure legends. All experiments could be successfully replicated and showed comparable results.
Randomization	For cell culture experiments, no randomization was necessary since cells were split, counted and plated before each experiments and then treated with DMSO/drugs or fluorescence probes so that equal amounts of cells and comparable conditions were assured in all wells
Blinding	For in vitro experiments, blinding was difficult to apply, since experiments were performed by the same investigator. However, all experiments were independently performed multiple times to ensure careful interpretation of the results.

Reporting for specific materials, systems and methods

We require information from authors about some types of materials, experimental systems and methods used in many studies. Here, indicate whether each material, system or method listed is relevant to your study. If you are not sure if a list item applies to your research, read the appropriate section before selecting a response.

Materials & experimental systems

n/a	Involved in the study	
☐	☒	Antibodies
☐	☒	Eukaryotic cell lines
☒	☐	Palaeontology and archaeology
☒	☐	Animals and other organisms
☒	☐	Human research participants
☒	☐	Clinical data
☒	☐	Dual use research of concern

Methods

n/a	Involved in the study	
☒	☐	ChIP-seq
☒	☐	Flow cytometry
☒	☐	MRI-based neuroimaging

Antibodies

Antibodies used	Antibodies were from Cell Signaling (9452 4E-BP1, 39749 SQSTM1(D1Q5S), 3724 HA-tag (C29F4), 2920 AKT (pan) (40D4), 4060 AKT pS473 (D9E), 12238 Calreticulin (D3E6), 13192 Golgin-97 (D8P2K), 4357 RagA (D8B5), 2280 Raptor (24C12), 9476 Rictor (D16H9), 2708 S6K (49D7), 9234 S6K pT389 (108D2), 8054 ULK1 (D8H5), 6888 ULK1 pS757, 2217 S6 (5G10), 2215 S6 pS240/244, 4661 VDAC (D73D12), 12994 ATG5 (D5F5U), 2276 myc-tag (9B11)) R&D Systems (AF965 cathepsin B, AF1515 cathepsin L, AF1029 cathepsin D,

AF1033 cathepsin X/Z/P), Invitrogen (MA3-026 CCT1(91A)), Sigma Aldrich (A5441 β-actin, PA22129 Pex19), GenScript (A00187-200 Flag-tag), Proteintech (15280 ATP6V1E1), Origene (TA802519 GAPDH(OTI2D9)) and Abcam (ab25245 Lamp1 (1D4B), ab202899 ATP6V0d1 (EPR18320-38), ab199326 ATP6V1A (EPR19270), ab92746 CCT2 (EPR4084). The LC3 antibody was a kind gift from Tullia Lindsten (MSKCC, NY). Secondary antibodies were from Life Technologies (31402 HRP-linked anti-goat), Cytiva (NA931 Amersham ECL Mouse IgG, NA934 Amersham ECL Rabbit IgG), and Sigma Aldrich (A10549 HRP-linked anti-rat). Primary antibodies were used at 1:1000 dilution, secondary antibodies at 1:5000 dilution.

Validation

Validation by ectopic target protein expression followed by western blotting:
HA-tag (C29F4) (Cell Signalling 3724) in Suppl. Fig. 6a.
Flag-tag (GenScript A00187-200) in Suppl. Fig. 6a.
Myc-tag (9B11) (Cell Signalling 2276) in Suppl. Fig. 7l.
Validation by CRISPR-mediated knockout followed by western blotting:
CCT1 antibody (91A) (Invitrogen MA3-026) in Fig. 5e.
CCT2 antibody ((EPR4084) (Abcam ab92746) in Fig. 5e.
Atg5 antibody (Cell Signalling 12994) in Suppl. Fig. 1j.
Cathepsin B antibody (R&D Systems AF965), Cathepsin L antibody (R&D Systems AF1515), Cathepsin D antibody (R&D Systems AF1029) in Suppl. Fig. 1c.
Other antibodies were validated by the respective manufacturers.
Cell Signalling:
9452 4E-BP1: https://www.cellsignal.de/products/primary-antibodies/4e-bp1-antibody/9452
39749 D1Q5S SQSTM1: https://www.cellsignal.de/products/primary-antibodies/sqstm1-p62-d1q5s-rabbit-mab/39749
2920 40D4 AKT (pan): https://www.cellsignal.de/products/primary-antibodies/akt-pan-40d4-mouse-mab/2920
4060 D9E AKTpS473: https://www.cellsignal.de/products/primary-antibodies/phospho-akt-ser473-d9e-xp-rabbit-mab/4060
12238 D3E6 Calreticulin: https://www.cellsignal.de/products/primary-antibodies/calreticulin-d3e6-xp-rabbit-mab/12238
13192 D8P2K Golgin-97: https://www.cellsignal.de/products/primary-antibodies/golgin-97-d8p2k-rabbit-mab/13192
4357 D8B5 RagA: https://www.cellsignal.de/products/primary-antibodies/raga-d8b5-rabbit-mab/4357
2280 24C12 Raptor: https://www.cellsignal.de/products/primary-antibodies/raptor-24c12-rabbit-mab/2280
9476 D16H9 Rictor: https://www.cellsignal.de/products/primary-antibodies/rictor-d16h9-rabbit-mab/9476
2708 49D7 S6K: https://www.cellsignal.de/products/primary-antibodies/p70-s6-kinase-49d7-rabbit-mab/2708
9234 108D2 S6KpT389: https://www.cellsignal.com/products/primary-antibodies/phospho-p70-s6-kinase-thr389-108d2-rabbit-mab/9234
8054 D8H5 Ulk1: https://www.cellsignal.de/products/primary-antibodies/ulk1-d8h5-rabbit-mab/8054
6888 ULK1pS757: https://www.cellsignal.de/products/primary-antibodies/phospho-ulk1-ser757-antibody/6888
2217 5G10 S6: https://www.cellsignal.de/products/primary-antibodies/s6-ribosomal-protein-5g10-rabbit-mab/2217
2215 S6 pS240/244: https://www.cellsignal.de/products/primary-antibodies/phospho-s6-ribosomal-protein-ser240-244-antibody/2215
4661 D73D12 VDAC: https://www.cellsignal.de/products/primary-antibodies/vdac-d73d12-rabbit-mab/4661
AF1033 mouse cathepsin X/Z/P: https://www.rndsystems.com/products/mouse-cathepsin-x-z-p-antibody_af1033
A5441 AC-15 β-actin: https://www.sigmaaldrich.com/DE/en/product/sigma/a5441
PA22129 Pex19: https://www.thermofisher.com/antibody/product/PEX19-Antibody-Polyclonal/PA5-22129
15280 ATP6V1E1: https://www.ptglab.com/products/ATP6V1E1-Antibody-15280-1-AP.htm
TA802519 OTI2D9 GAPDH: https://www.origene.com/catalog/antibodies/primary-antibodies/ta802519/gapdh-mouse-monoclonal-antibody-clone-id-oti2d9
ab25245 1D4B Lamp1: https://www.abcam.com/lamp1-antibody-1d4b-ab25245.html
ab202899 EPR18320-38 ATP6V0D1: https://www.abcam.com/atp6v0d1p39-antibody-epr18320-38-ab202899.html
ab199326 EPR19270 ATP6V1A: https://www.abcam.com/atp6v1a-antibody-epr19270-ab199326.html

Eukaryotic cell lines

Policy information about cell lines

Cell line source(s)	Human MIA PaCa-2 (ATCC CRL-1420), HEK 293T (ATCC CRL-3216), MRC-5 (ATCC CCL-171), A549 (ATCC CCL-185), SV40 large T-immortalized MEFs (Palm et al., Cell 2015).
Authentication	All used human cell lines were authenticated by short tandem repeat profiling or sequencing analysis on the 24-02-2021
Mycoplasma contamination	All used cell lines were routinely tested and confirmed negative for mycoplasma contamination.
Commonly misidentified lines (See ICLAC register)	No commonly misidentified cell lines were used.
